# Validation of a Dutch version of the Geriatric Oral Health Assessment Index (GOHAI-NL) in care-dependent and care-independent older people

**DOI:** 10.1186/s12877-016-0227-0

**Published:** 2016-02-29

**Authors:** Dominique Niesten, Dick Witter, Ewald Bronkhorst, Nico Creugers

**Affiliations:** Department of Oral Function, College of Dental Sciences, Radboud University Nijmegen Medical Center, PO Box 9101HB, Nijmegen, The Netherlands; Department of Oral Function, College of Dental Sciences, Radboud University Nijmegen Medical Center, Nijmegen, The Netherlands; Department of Cariology and Preventive Dentistry, College of Dental Sciences, Radboud University Nijmegen Medical Center, Nijmegen, The Netherlands

**Keywords:** GOHAI, GOHAI-NL, Validation, Oral heath-related quality of life, Gerodontology, Dutch

## Abstract

**Background:**

The GOHAI is a frequently used instrument to measure oral health-related quality of life (OHRQoL) of adults, in particular older people. The aim of this study was to translate the original English version of the GOHAI into a Dutch version (GOHAI-NL), and to test the validity and reliability of the GOHAI-NL in care-independent and care-dependent older people.

**Methods:**

The GOHAI questionnaire was translated into Dutch, discussed by an expert panel, back-translated to the original, pilot-tested and assessed for cognitive and conceptual equivalence. The resulting GOHAI-NL was tested in a groups of care-independent (Group A, *n* = 109, mean age 73.1 ± 5.4 years) and care-dependent (Group B, *n* = 118, mean age 85.6 ± 7.0. years) cognitively alert people of 65 years and over. Psychometric properties including reliability (internal consistency, item-total, item-dimension, dimension-total, inter-item correlation, and test-retest stability), and validity (convergent, discriminant, known-group), and floor and ceiling effects were assessed.

**Results:**

Internal consistency was confirmed by Cronbach’s alphas of 0.86 (group A) and 0.80 (group B). Item-total score correlations were between 0.4 and 0.7 except for item 3 in group A (0.34) and B (0.08) and for item 12 in group A (0.20). Item-dimension and dimension-total correlations were between 0.30 and 0.78 and around 0.7 respectively for the dimensions ‘physical functioning’ and ‘psychosocial functioning’, but lower for the dimension ‘pain and discomfort’ with item-dimension correlations between 0.13 and 0.44. Average inter-item correlations were 0.34 ± 0.11 (group A) and 0.33 ± 0.08 (group B). Test-retest correlation of the total score (GOHAI-ADD) was 0.88 in group A (ICCs: 0.62 - 0.88) and 0.93 in group B (ICCs: 0.64 – 0.91). Significant correlations in the expected direction were found between GOHAI and most oral health-related variables except for presence of caries in group A, and perceived general health, prosthodontic status and number of natural teeth in group B. No floor or ceiling effects were detected at GOHAI-ADD level; however ceiling effects did occur at dimension level.

**Conclusion:**

The GOHAI-NL has satisfactory reliability and validity and can be used to measure OHRQoL in Dutch care-dependent and care-independent older people.

**Electronic supplementary material:**

The online version of this article (doi:10.1186/s12877-016-0227-0) contains supplementary material, which is available to authorized users.

## Background

A range of instruments that measure oral health-related quality of life (OHRQoL) has been developed in the last two decades. [1]. One of these instruments is the Geriatric Oral Health Assessment Index (GOHAI), a frequently used questionnaire that aims to assess OHRQoL within older populations [2]. It comprises of 12 items that measure three dimensions of OHRQoL: physical function (3 items), psychosocial function (5 items) and pain/discomfort (4 items).

Several studies indicate that the GOHAI is a more suitable instrument to measure OHRQoL of the elderly in Western cultures than the currently most frequently used Oral Health Impact Profile (OHIP) [3–7]. The OHIP taps more severe OHRQoL impacts than the GOHAI and is generally less sensitive to minor impairment of OHRQoL [3]. As a consequence larger proportions of participants report no impact, i.e. have zero-scores (floor effect) when using the OHIP than when using the GOHAI [3, 8]. Based on epidemiological data this floor effect is likely to also occur for Dutch elderly [9, 10]. This effect reduces the ability of the OHIP to detect within-subject changes, when compared with the GOHAI. However, no validated Dutch version of the GOHAI is available.

The aim of this study was to translate the original English version of the GOHAI into a Dutch version (GOHAI-NL), and to validate the translated instrument for use in epidemiological surveys among older people in the Netherlands. To warrant validation for a wide spectrum of older people, we chose to validate the GOHAI for both severely frail, care-dependent older people and for care-independent older people.

Although the GOHAI was originally intended as a self-administered questionnaire, this administration method is likely to generate unreliable results in severely frail and care-dependent older people who often have impairments (e.g. visual, cognitive) that affect their capacity to complete self-administered questionnaires [11, 12]. Therefore, for care-dependent older people we chose to administer the GOHAI questionnaire through a personal interview.

## Methods

### Translation

The original GOHAI questionnaire [2] was independently translated into Dutch by two bilingual translators whose native language was Dutch. One of them was a dental researcher experienced in the use of quality of life measures (DN), the other was a professional translator specialized in the translation of patient reported outcome measures. We adhered to the “Principles of Good Practice for the Translation and Cultural Adaptation Process for Patient-Reported Outcomes Measures” [13]. The two forward-translations were reconciled into one forward translation by an expert panel consisting of a dentist-researcher, a geriatric dentist-researcher and an oral health researcher. Competing options were discussed, and other bilingual experts were consulted when necessary, until consensus was reached. The resulting forward-translation was independently back-translated into English by two professional translators whose native language was English. The back-translations were compared for conceptual equivalence with the original GOHAI by the expert panel. Problematic items were identified and discussed among the expert panel and with the translators. Based on their comments, the forward-translation was refined. The resulting translation was tested in a purposive sample consisting of 10 older (65 years and over) people whose self-reported general health was bad (*n* = 3), mediocre (*n* = 3), or good (*n* = 4). The translation was tested for cognitive equivalence and comprehensibility. Based on received comments, the translation was finalized by the expert panel.

### Respondent selection

In order to test and validate the proposed GOHAI-NL, participants of 65 years and over were recruited from two independent samples (group A and group B).

These two groups were recruited in order to represent distinct differences in frailty and general health within the population of elderly. Group A represented non-frail care-independent older people with expected good health and group B represented frail care-dependent, but cognitively alert people with compromised health.

Because gender and dental/prosthodontic status are known to possibly influence self-perceived oral health, for both groups an even distribution of men and women, and of (partially) dentulous (having at least one natural tooth) and edentulous (with or without complete removable dental prostheses (CRDP’s)) participants was sought [14–17].

Participants of group A were recruited in the clinic of the Dental School of Radboud University Medical Center through convenience sampling, and comprised of independent living, cognitively alert subjects who came for periodical check-up visits between 2013 and 2015. Since this sample was recruited from a generally healthy, independently living population with no registered health impairments according to the patients’ dental records, it was assumed that the chance of recruits being frail would be small. Upon provision of informed consent, after their clinical examination, they were asked to complete a questionnaire, including the GOHAI-NL.

Participants of group B were recruited in a total of 11 residential aged care facilities (RACFs), selected through convenience sampling, in the southern part of the Netherlands. The RACFs were included after the management’s consent to have their residents examined on a voluntary base. The care managers of the RACF’s recruited the participants for this study, based on instructions by the principal researcher (DN). These instructions included exclusion of subjects who were not cognitively alert according to the responsible ward nurse. All participants in group B had a certain level of care dependency as determined by a medical authority, based on the Dutch care-dependency classification system (Dutch National Centre for Indication of Care Need (CIZ; www.ciz.nl)). Each RACF resident is assigned a ‘Package of Care Dependency’ according to this system, indicating the level and type of care needed referring to impairments in the physical and/or mental and/or social domain.

Upon provision of their informed consent, the participants received a clinical examination by a final year dental student or a final year dental hygiene student. Next, they were personally interviewed by the principal researcher who used the same questionnaire as the one used for group A.

Convenience quota sampling was used aiming at a total sample size of approximately 120 recruits for each group. Sample size was calculated based on the recommendation to include 5–10 subjects per questionnaire item [18], resulting in a need for 60–120 participants per group.

### Data

Participants were asked to provide information regarding their oral health by answering the GOHAI questionnaire and four additional questions: *1. How do you perceive your oral health (very bad, bad, moderate, good, very good); 2. Are you satisfied with your oral health (y/n); 3. Do you think you need dental treatment at the moment (y/n); 4. How do you perceive your general health (very bad, bad, moderate, good, very good).* The GOHAI questionnaire includes 12 questions (each question addressing one oral health item). Respondents were asked how often, in the previous three months, they have experienced the oral health item addressed: ‘never’, ‘seldom,’ ‘sometimes’, ‘often’, or ‘very often or always’. Besides, date of birth, gender, and nationality were recorded. Socio-economic status (SES) (high, middle, low) was assessed based on last held occupation (according to the ISCO-08 classification [19]) and on level of education (high, middle, low); the highest level of either education or occupation determined SES.

Clinical data were obtained through examinations by calibrated final year dental students (all kappa’s > 0.82; overall κ =0.87; agreement = 90.1 %) or calibrated final year dental hygiene students (all kappa’s > 0.66; overall κ =0.74; agreement = 84.4 %). Data included number and position of 1) natural teeth, 2) caries lesions, 3) restorations (such as direct restorations or fixed dental prostheses), and 4) partial or complete removable dental prostheses. The WHO criteria for assessment of the aforementioned variables were used [20]. In addition, clinical treatment need (y/n) was recorded, based on the clinically assessed need for any professional dental treatment including reline, rebase or replacement removable dental prostheses.

Group A participants were examined in the clinic of the dental school while group B participants received a clinical examination at their residence, where the examiners used hand held torches and a dental mirror.

### Missing data

Participants with two or more GOHAI answers missing, or with one or more answers to the additional questions missing, or with missing clinical data were excluded. In case only one GOHAI answer was missing, the missing value was replaced by mean substitution.

In case clinical data were recorded more than two weeks before or after the questionnaire was completed, the participant was excluded. This was done in order to minimize the chance that the clinical status of the participant was different from that at the moment of completing the questionnaire.

### Analyses

#### General psychometric properties

Answer proportions (%) of each of the GOHAI-NL items and of the GOHAI-ADD (additive) score and the GOHAI-SC (simple count) score [2] were calculated. The GOHAI-ADD score is the sum of all scores (score 1 to 5 per answer; total score from 12 to 60). The GOHAI-SC score is the sum of all items with response ‘sometimes’, ‘often’ and ‘always or nearly always’ (score 0 or 1 per answer; total score from 0 to 12), where a ‘1’-score indicates impairment for that item [2]. Item scores for questions 3, 5, and 7 were reverse-coded so that all items scored in the same direction; higher values indicating better OHRQoL.

Floor and ceiling effects were assessed at dimension level (with GOHAI dimensions: physical function, pain and discomfort, and psychosocial function), and at total score level (GOHAI-ADD; GOHAI-SC). Floor and ceiling effects were considered present when 20 % or more participants had the lowest (floor) or highest (ceiling) possible total score [21].

#### Reliability

Reliability was assessed by measuring internal consistency and stability. Internal consistency was measured through correlation between item scores and the overall GOHAI-ADD score, using the corrected item-total score correlation (Spearman’s rank correlation coefficient) and Cronbach’s alphas. Overall Cronbach’s alphas > 0.7 and > 0.9 are considered indicative for acceptable consistency for comparisons at group level and at individual level, respectively [22, 23]. The dimensional structure of the GOHAI-NL was evaluated through assessment of correlations between item scores and the GOHAI-ADD score of the related dimension (subscale). Cronbach’s alphas > 0.4 are considered indicative for adequate item - subscale consistency and Cronbach’s alphas > 0.7 are considered indicative for adequate subscale - overall scale (total score) consistency [22]. Inter-item correlations were calculated in order to determine the extent to which the items were related to each other (average inter-item correlation ideally should be between 0.2 and 0.5 [23, 24]), and to detect redundancy of items (Cronbach’s alpha > 0.7) [25].

Stability was assessed by measuring test-retest reliability through calculation of intraclass correlation coefficients (ICCs) (two-way mixed, single measure) in two subsamples consisting of randomly selected respondents from group A and group B. Participants of these samples groups were either sent a second questionnaire (group A) or interviewed a second time (group B) after one to two weeks after they had returned their first questionnaire or were interviewed, as it was expected that no major differences in oral status and oral health would have occurred during this time interval. ICC values > 0.75 were considered indicative for excellent stability, 0.40 – 0.75 for fair to good, and < 0.40 for poor stability [26].

#### Validity

Validity was measured through convergent validity, discriminant validity, and known-group validity. Convergent validity refers to the degree to which two measures that should measure the same construct, are related. This was determined through assessment of the correlations between GOHAI-ADD scores and the answers to two general questions on self-perceived oral health: 1. How do you perceive your oral health; 2. Are you satisfied with your oral health.

Discriminant validity refers to the degree to which two measures that should measure two similar, but conceptually different constructs are related. This was determined through the correlation between the GOHAI-ADD scores and 1. clinical treatment need; 2. presence of caries lesions; and 3. self-perceived general health. A low to moderate correlation was expected between higher GOHAI-ADD scores on the one hand and less clinical treatment need, absence of caries lesions, and better self-perceived general health on the other.

Known-group validity refers to the degree to which a measure is sensitive to differences within subgroups that are assumed to be reflected in the scores. This was assessed by comparing differences in GOHAI-ADD scores between subgroups with different self-perceived treatment need (y/n), a higher number of natural teeth, and different dental / prosthodontic status (natural teeth without removable dental prostheses (RDPs), natural teeth with partial or complete RDPs, or no natural teeth (with or without complete RDPs)). Participants without self-perceived treatment need, with higher numbers of natural teeth, and without removable dental prostheses, were assumed to have higher GOHAI-ADD scores.

Correlations were assessed by calculating Spearman’s rank correlation coefficients (r), with values > 0.5 indicating a strong correlation, 0.35 to 0.5 a moderate correlation, and 0.2 to 0.34 a low correlation [27, 28].

#### Ethics, consent and permissions

The study was approved by the Medical Ethics Committee (CMO) of the Radboud University Medical Center Nijmegen (CMO ref. 2012/294). All participants were informed (in writing and personally) about the study and provided written consent prior to their participation.

## Results

### Translation

Translation procedures and discussions among the expert panel yielded no irresolvable issues concerning semantic, experiental or conceptual equivalence. The resulting GOHAI-NL is presented in Additional file 1.

### Characterization of groups and subjects

The original sample consisted of 232 participants; 111 in group A and 121 in group B. After exclusion of subjects because of 2 or more missing GOHAI answers (group A; *n* = 2) or missing clinical data (Group B; *n* = 3) respectively, group A included 109 participants and group B 118 (Table 1). For two participants in group A who missed one GOHAI question, the mean substitution was imputed. In group A, 47.7 % of the participants were female; 60.6 % were dentate (at least one natural tooth) and the mean age was 73.1 ± 5.4. In group B, 57.6 % were female; 49.2 % were dentate and the mean age was 85.6 ± 7.0. Group A participants had a slightly higher SES (high 31.9 %, medium 50.5 %, low 17.8 % versus high 23.9 %, medium 40.2 %, low 35.9 % in group B).

**Table 1:** Sample Characteristics and Analyses per Sample

Sample	Number	Age Mean (SD)	Gender %female	Dentate^a^ %	Data administration	Analyses
Group A: Independent living dental clinic attenders of 65+ yrs (check-up visits)	109	73.1 (5.4)	47.7	60.6	questionnaire	general psychometric properties, floor and ceiling effects, internal consistency (item-total scale, dimension-total scale, inter-item), convergent, discriminant, and known-group validity
Group B: Institutionalized care-dependent elderly of 65+ yrs	118	85.6 (7.0)	57.6	49.2	personal interview	general psychometric properties, floor and ceiling effects, internal consistency (item-scale, dimension-scale, inter-item), convergent, discriminant, and known-group validity
subsample (convenience sample) of group A	32	74.0 (5.8)	50.0	78.1	questionnaire	test-retest reliability
subsample (convenience sample) of group B	34	85.9 (6.9)	47.1	50.0	personal interview	test-retest reliability

^a^minimum of 1 natural tooth

### General psychometric properties

Answer proportions and percentage impairment (based on the number of answers ‘sometimes’, ‘often’ or ‘nearly always or always’) for group A and B are listed in Table 2. The mean GOHAI-ADD score was 51.5 ± 7.5 (range 29–60) for group A and 52.4 ± 6.1 (range 26–60) for group B. Mean GOHAI-SC score was 1.9 ± 2.4 (range 0–9) for group A and 1.9 ± 1.9 (range 0–9) for group B. The items that showed highest frequency of impairment were item 9 (30.2 %), item 2 (28.5 %), and item 5 (23.8 %) for group A and item 2 (48.3 %), item 7 (39.8 %), and item 5 (26.3 %) for group B, indicating that most impairment was reported in relation to oral function (items 1, 2 and 4) and psychological aspects (items 6, 7, 9, 10, 11) (Table 2). The items that showed lowest frequency of impairment were items 6, 8, and 10 for both groups, indicating that least impairment was reported in relation to psychosocial aspects, which was emphasized by the zero scores in answer categories ‘often’ and ‘nearly always or always’ of items 6, 9, 10, and 11.

**Table 2:** Answer proportions and percentage participants scoring ‘impairment’^a^ per GOHAI item for groups A and B^b^

GOHAI item	group	never	seldom	sometimes	often	very often or always	% impair-ment
1. Limit the kinds of food	A	64.2	14.7	10.1	6.4	4.6	21.1
	B	61.9	16.1	11.9	5.9	4.2	22.0
2. Trouble biting or chewing	A	39.4	32.1	13.8	8.3	6.4	28.5
	B	34.7	16.9	14.4	22.9	11	48.3
3. Able to swallow comfortably	A	7.3	5.5	5.5	19.3	62.4	18.3
	B	7.6	2.5	5.9	15.3	68.6	16.0
4. Unable to speak clearly	A	70.6	18.3	7.3	0.9	2.8	11.0
	B	77.1	5.9	11.9	2.5	2.5	16.9
5. Able to eat without discomfort	A	6.4	10.1	7.3	24.8	51.4	23.8
	B	2.5	8.5	15.3	28.0	45.8	26.3
6. Limit contact with people	A	86.2	10.1	1.8	0.9	0.9	3.6
	B	93.2	5.9	0.8	0.0	0.0	0.8
7. Pleased with look of teeth	A	3.7	4.6	8.3	56.9	26.6	16.6
	B	11	14.4	14.4	29.7	30.5	39.8
8. Used medication to relieve pain	A	70.6	21.1	7.3	0.9	0.0	8.2
	B	93.2	3.4	1.7	0.8	0.8	3.3
9. Worried about teeth, gums or dentures	A	37.6	32.1	16.5	12.8	0.9	30.2
	B	65.3	16.9	15.3	2.5	0.0	17.8
10. Self-conscious of teeth, gums or dentures	A	67.9	21.1	9.2	0.0	1.8	11.0
	B	84.7	9.3	5.9	0.0	0.0	5.9
11. Uncomfortable eating in front of others	A	70.6	14.7	9.2	3.7	1.8	14.7
	B	82.2	11.0	5.9	0.8	0.0	6.7
12. Sensitive to hot, cold or sweet foods	A	47.7	32.1	15.6	3.7	0.9	20.2
	B	68.6	9.3	13.6	7.6	0.8	22.0

^a^combined answers ‘sometimes’,‘often’, and ‘very often or always’; reverse coded for items 3, 5, 7

^b^Group A: care-independent subjects, *n* = 109; Group B: care-dependent subjects, *n* = 118

No floor or ceiling effects were detected at total score level (GOHAI-ADD): 7.3 % (group A) and 12.7 % (group B) had the highest possible score of 60, none had the lowest possible score of 12. The GOHAI-SC score however did show a floor effect: 42.2 % of group A participants and 28.0 % of group B participants had a total score of zero. At dimension level, there were no floor effects. However, ceiling effects occurred in two dimensions in group A and in all 3 dimensions in group B. Maximum scores were obtained by 37.6 % (physical function), 21.1 % (pain and discomfort) and 17.4 % (psychosocial function) of group A participants; and by 28.0 % (physical function), 28.8 % (pain and discomfort), and 28.0 % (psychosocial function) of group B participants.

### Reliability

Cronbach’s alphas were 0.86 for group A and 0.80 for sample B, indicating good overall internal consistency. The corrected item-total score correlations were between 0.4 and 0.7 indicating adequate correlation, except for item 3 in both group A (r = 0.34) and group B (r = 0.08), and for item 12 in group A (r = 0.20) (Table 3).

**Table 3:** Reliability analysis based on item-total score correlation and test-retest correlation

GOHAI item	Corrected Item-Total score Correla-tion	Cronbach's Alpha if Item Deleted	Test-retest correla-tion ICC^a^	Corrected Item-Total score Correla-tion	Cronbach’s Alpha if Item Deleted	Test-retest correla-tion ICC^a^
	Group A			Group B		
1. Limit the kinds of food	0.65	0.84	0.75	0.61	0.77	0.91
2. Trouble biting or chewing	0.67	0.84	0.81	0.53	0.78	0.91
3. Able to swallow comfortably	0.34	0.86	0.62	0.08	0.83	0.74
4. Unable to speak clearly	0.63	0.84	0.86	0.40	0.79	0.94
5. Able to eat without discomfort	0.49	0.85	0.78	0.67	0.77	0.74
6. Limit contact with people	0.43	0.85	0.64	0.47	0.80	0.79
7. Pleased with look of teeth	0.62	0.84	0.68	0.58	0.78	0.69
8. Used medication to relieve pain	0.51	0.85	0.74	0.53	0.79	0.81
9. Worried about teeth, gums or dentures	0.60	0.84	0.80	0.51	0.78	0.64
10. Self-conscious of teeth, gums or dentures	0.69	0.84	0.79	0.52	0.79	0.73
11. Uncomfortable eating in front of others	0.74	0.83	0.88	0.61	0.78	0.64
12. Sensitive to hot, cold or sweet foods	0.20	0.87	0.73	0.43	0.79	0.82

^a^ICC Intraclass correlation coefficient; applied to subsamples of group A (*n* = 32) and B (*n* = 34)

Inter-item correlations were within the acceptable range of 0.2–0.5 for both groups (mean Cronbach’s α group A: 0.34 ± 0.11; mean Cronbach’s α group B: 0.33 ± 0.08). Inter-item correlations > 0.7 occurred only in group A, between items 1 and 2 (Cronbach’s α = 0.76) and between items 10 and 11 (Cronbach’s α = 0.74); indicating possible redundancy.

Test-retest reliability (stability) was high for both groups: mean 0.88 (GOHAI-ADD) and 0.87 (GOHAI-SC) for group A, and 0.93 (GOHAI-ADD) and 0.89 (GOHAI-SC) for group B. ICCs of 0.62 - 0.88 in group A and 0.64 – 0.91 in group B indicated overall good stability, with least stability for items 3, 6 and 7 in group A, and for items 7, 9, and 11 in group B (Table 3).

The dimensional structure of the orginal GOHAI was only partly supported by Cronbach’s alphas and item - subscale correlation values (Table 4). Cronbach’s alphas for subscale - overall scale correlation were around the treshold of 0.7 for the dimensions ‘physical functioning’ and ‘psychosocial functioning’, and all item - subscale correlations within these dimensions were adequate (above > 0.45) except for item 4, ‘trouble speaking clearly’, in group B. Items within the dimension ‘pain and discomfort’ (items 3, 5, 8, 12) were only weakly correlated to the dimension total score (Cronbach’s alphas between 0.13 and 0.44). This dimension showed inadequate (<0.7) subscale - overall scale consistency in both groups A and B.

**Table 4:** Correlation between item - subscale (dimension) scores and between subscale- overall scale scores

GOHAI items and dimension	Group	Cronbach’s alpha
Dimension: Physical Functioning subscale-overall scale Cronbach's α: group A: 0.82; group B: 0.64		
1. Limit the kinds of food	A	0.78
	B	0.54
2. Trouble biting or chewing	A	0.81
	B	0.55
4. Unable to speak clearly	A	0.49
	B	0.30
Dimension: Pain and discomfort subscale-overall scale Cronbach's α group A: 0.43; group B: 0.49		
3. Able to swallow comfortably	A	0.31
	B	0.19
5. Able to eat without discomfort	A	0.31
	B	0.36
8. Used medication to relieve pain	A	0.26
	B	0.44
12. Sensitive to hot, cold or sweet foods	A	0.13
	B	0.28
Dimension: Psychosocial functioning subscale-overall scale Cronbach's α group A: 0.82; group B: 0.72		
6. Limit contact with people	A	0.46
	B	0.46
7. Pleased with look of teeth	A	0.65
	B	0.59
9. Worried about teeth, gums or dentures	A	0.52
	B	0.65
10. Self-conscious of teeth, gums or dentures	A	0.76
	B	0.59
11. Uncomfortable eating in front of others	A	0.76
	B	0.48

### Validity

Table 5 shows the main results of comparisons between assumedly construct-related variables and GOHAI-ADD scores.

**Table 5:** Validity assessments: Spearman’s rank correlations between selected variables and GOHAI-ADD scores

Type of validity		Group A			Group B		
Variable	Answer categories	*n*	Mean GOHAI-ADD score (SD)	Correlation (r), *p*-value	*n*	Mean GOHAI-ADD score (SD)	Correlation (r), *p*-value
Convergent validity							
Perceived oral health							
Very bad		0	-	*r* = 0.42	2	31.50 (7.78)	*r* = 0.68
Bad		1	31.00 (-)	*p* <0.001	13	44.46 (6.67)	*p* <0.001
Moderate		23	46.39 (8.47)		23	49.30 (5.66)	
Good		83	53.48 (6.13)		53	53.64 (4.19)	
Very good		2	59.00 (0.00)		26	57.81 (2.58)	
Satisfied with oral health							
Yes		93	53.63 (6.00)	*r* = 0.47	84	54.71 (4.54)	*r* = 0.52
No		15	41.73 (7.75)	*p* <0.001	34	46.32 (7.24)	*p* <0.001
Discriminant validity							
Perceived general health							
Very bad		NA	NA	*r* = 0.24	3	52.00 (8.54)	*r* = 0.10
Bad		1	59.00 (-)	*p* = 0.014	24	51.21 (7.66)	*p* = 0.30
Moderate		21	47.62 (9.46)		38	52.05 (6.32)	
Good		74	52.45 (6.88)		49	52.88 (6.54)	
Very good		11	55.64 (4.99)		4	54.25 (4.27)	
Clinical treatment need							
Yes		40	48.80 (8.56)	*r* = 0.29	65	50.52 (6.36)	*r* = 0.36
No		69	53.67 (6.24)	*p* = 0.002	53	54.47 (6.34)	*p* = <0.001
At least one tooth with caries^a^							
Yes		7	51.86 (6.09)	*r* = 0.08	32	50.66 (5.88)	*r* = 0.42
No		59	53.15 (6.00)	*p* = 0.55	26	55.15 (5.53)	*p* = 0.001
Known-group validity							
Dental/ prosthodontic status							
Natural teeth without RDP		44	54.36 (4.62)	*r* = 0.29	24	53.67 (6.29)	*r* = 0.07
Natural teeth with RDP		22	50.68 (7.42)	*p* = 0.003	34	51.94 (5.97)	*p* = 0.44
No natural teeth		43	49.12 (8.84)		60	51.95 (7.13)	
no. of natural teeth^a^ (1-32)		66		*r* = 0.39	58		*r* = 0.24
				*p* <0.001			*p* = 0.067
Perceived need for treatment							
Yes		45	48.11 (8.24)	*r* = 0.48	33	46.33 (7.50)	*r* = 0.53
No		63	54.79 (5.31)	*p* <0.001	85	54.61 (4.51)	*p* <0.001
Other correlations							
Gender							
Female		52	50.33 (8.01)	*r* = 0.17	68	52.40 (6.76)	*r* = 0.01
Male		57	53.30 (6.80)	*p* = 0.08	50	52.16 (6.50)	*p* = 0.88
age	(65–100)			*r* = 0.09			*r* = 0.09
				*p* = 0.34			*p* = 0.36
SES	high	34	53.00 (5.96)	*r* = -0.18	28	54.21 (5.42)	*r* = -0.14
	middle	54	51.39 (7.72)	*p* = 0.07	47	52.11 (7.27)	*p* = 0.13
	low	19	48.79 (8.66)		42	51.33 (6.53)	

^a^subjects with at least 1 natural tooth

*r Spearman’s rank correlation coefficient

Convergent validity: moderate to high (0.42–0.68), significant correlations in the expected direction were found between GOHAI-ADD scores for self-perceived oral health and satisfaction with oral health for both groups A and B.

Discriminant validity: low to moderate (0.24–0.42), but significant correlations in the expected direction were found between GOHAI-ADD scores and self-perceived general health (group A), clinical treatment need (group A and B) and presence of caries (group B). Non-significant were the correlations between self-perceived general health (group B), and presence of caries (group A); these correlations found were, however, in the expected direction.

Known-group validity: moderate, significant correlations in the expected direction (group A: r = 0.48; group B: r = 0.53) were found between GOHAI-ADD scores and self-perceived treatment need. GOHAI-ADD scores were also significantly correlated in the expected direction for dental / prosthodontic status (r = 0.29) and number of natural teeth (r = 0.39) for group A, but not for group B.

Differences in age, gender and SES were not statistically significantly correlated with GOHAI-ADD; however higher SES levels were correlated with higher GOHAI-ADD scores in both groups (Table 5).

## Discussion

### Study design

This study tested psychometric properties of a Dutch version of the GOHAI, including validity and reliability. The original GOHAI was validated in a population of older well-educated Americans. Although the GOHAI has been demonstrated to also be valid for younger and for less educated population samples [29, 30], it remains important that validity problems related to differences in language or culture are ruled out. This is why we undertook an evidenced approach [13] to assure conceptual equivalence between the GOHAI-NL and the original GOHAI.

Following the vast majority of GOHAI validation studies, we calculated GOHAI-SC scores in addition to the standardly used GOHAI-ADD scores. Although the use of GOHAI-SC scores implies some loss of information because it requires dichotomization of GOHAI answers, the GOHAI-SC provides a reliable, albeit crude, estimate of perceived oral impairments.

To our knowledge, our study is the first that validates the GOHAI in two distinct groups of older people using different administration methods. This choice was prompted by the evidence that the use of self-administered questionnaires in severely frail older populations does not always yield reliable results [31]. We therefore used personal interviews as the administration method in this group. When using personal interviews, any problems related to cognitive abilities of the respondent can be detected more easily. Although the GOHAI has been used in people with mild cognitive impairments [32, 33] it has not been validated for such populations. Therefore we do not recommend the use of the GOHAI-NL in cognitively impaired subjects except when closely related informants provide support in answering questions, and with explicit reference to this fact.

### Limitations

The administration method used in group B may have induced a degree of social desirability bias, leading to expectedly ‘too high’ scores. Reissmann [34] showed that OHIP outcomes obtained through personal interviews were 15 % lower (indicating less oral health-related complaints) than outcomes derived from self-administered questionnaires in a group of older adults. In the present study, we could not examine the effect of these two administration methods on GOHAI-scores because these methods were applied in different samples. It is recommended to compare the effects of different methods of administration on acquired GOHAI scores within groups of frail and non-frail older people in future research.

Our study did not measure responsiveness to change in oral health status of the GOHAI-NL and hence additional longitudinal research is recommended to assess the sensitivity of the GOHAI-NL for monitoring oral health changes*.*

### Results

In the translation procedure, the expert panel decided to use the Dutch equivalent of ‘very often or always’ instead of ‘always’ in the original version. This follows the reasoning used in the translation to the German GOHAI [5]: ‘always’ (‘altijd’) in Dutch is very strictly interpreted as ‘not a moment without’, and the distance between the alternative response options ‘often’ and ‘very often or always’ is expectedly more equal to the distances between other consecutive response options, as meant in a Likert-scale [35], than the (expectedly larger) distance between‘often’ and ‘always’.

The double negative phrasing of item 5 of the original GOHAI “how often were you able to eat *without discomfort*” has been documented to lead to inconsistent answers [30, 36]. In our study, item 5 had relatively low item-total correlation in group A and around 6 % of the anwers to the (self administered) item 5 were considered to be inconsistent with reference questions. The effect of double negative phrasing may be mitigated through adding reading notes to the questionnaire; which should be considered for all international GOHAI versions.

The mean GOHAI-ADD scores of 52.4 ± 7.5 in group A and of 52.5 ± 6.1 in group B in this study are similar to those found in Northwestern Europe and the USA (53 in Germany, 49.8 in Sweden, 46.4 in France, and 52.5 in the USA) [2, 30, 37, 38] but higher than those found elsewhere in the world (mean GOHAI-ADD scores between 18 and 49 in Romania Hongkong, Japan, Malaysian, Jordan, Turkey, India, Spain, Mexico, Iran, see also overview in Rezaei e.a. [39]). This is considered to be not only due to differences in oral health status, but also to variations in perceptions and expectations of oral health as well as in the self-reporting of oral health impacts, which are in part explained by cultural differences.

Although GOHAI outcomes of groups A and B are not meant to be compared because of the different administration methods used, the lack of difference between GOHAI-ADD scores is striking against the differences in clinically assessed oral health status between both groups (group B having worse oral health). The relatively high GOHAI scores of group B are most likely caused by social desirability bias (as addressed above) and by the so-called ‘disability paradox’ of older people that implies that they have better self-perceived oral health despite worse oral health status [40, 41].

Contrary to the OHIP [3, 6, 8], the GOHAI did not demonstrate floor and ceiling effects for the overall GOHAI-ADD score, which is the most used outcome measure for group comparisons of the GOHAI. At subscale (dimension) level, however, floor effects were detected. This means that the subscales are not capturing the full range of potential GOHAI responses in the population and that the ability to detect changes over time may be compromised [42].

Regarding reliability: both overall internal consistency (Cronbach’s alphas of 0.86 (group A) and 0.80 (group B)) and overall stability (Cronbach’s alphas of 0.88 (group A) and 0.93 (group B)) were good and comparable with values of other GOHAI studies [5, 8, 30, 37, 39, 43–46]. Items 3 (ability to swallow) and 12 (sensitivity to hot, cold and sweets) showed low correlation with the total GOHAI scores, which is in line with several previous validation studies [5, 30, 39, 47, 48]. Both items probably refer to a different construct than that intended to be measured by the GOHAI, which is oral health-related quality of life. One respondent in our study criticized item 12 saying that any human tissue is sensitive to hot and cold. Hence apart from the questionable conceptual correlation between teeth and tissue sensitivity and oral health, ambiguous interpretation of this item is likely to contribute to the found low item-total correlation.

The subscale (dimension) - overall scale correlation for the dimension ‘pain/discomfort’ was too low to justify distinction of this dimension. Since this finding is supported by ample evidence against the original dimensional structure of the GOHAI [5, 36, 37, 45, 49], it may be worthwile to reconsider these dimensions or opt for a one-dimensional scale.

Regarding validity: the GOHAI-NL was in good agreement with other measures of perceived oral health and demonstrated overall good convergent and adequate discrimant and known-group validity, supporting its construct validity. The low correlation between presence of carious lesions and GOHAI-ADD scores in the care-independent elderly was probably at least partly due to the low numbers of carious lesions encountered in group A, where only 7 out of 66 dentates had one or more carious teeth. The low correlations between GOHAI-ADD scores on the one hand and self-perceived general health and dental/prosthodontic status on the other that were found in this study in the group of care-dependent elderly, were unexpected. Although there is some evidence indicating that the correlation between general health and oral health is weaker in populations with impaired general health in comparison to healthy populations, generally the association between perceived oral health and perceived general health is strong [50, 51]. With regard to prosthodontic status, the lack of correlation, which is in contrast with findings from the majority, but not all GOHAI validation studies (e.g. [5, 30, 37, 44] vs. [39, 49]), may be due to the adaptation of frail elderly to oral discomfort caused by removable dental prostheses [52, 53].

## Conclusion

This study shows that the GOHAI-NL has satisfactory reliability and construct validity and can be used to measure OHRQoL in Dutch care-dependent and care-independent older people.

## Additional file

Additional file 1:
**GOHAI-NL.** (DOCX 11 kb)

## Abbreviations

GOHAI: geriatric oral health assessment index
GOHAI-ADD: geriatric oral health assessment index additive (score)
GOHAI-NL: Dutch version of the Geriatric Oral Health Assessment Index
GOHAI-SC: geriatric oral health assessment index simple count (score)
ICC: intraclass correlation coefficient
OHIP: oral health impact profile
OHRQoL: oral health-related quality of life
RACF: residential aged care facility
SES: socio-economic status
