# Supplementary material for: Validation of a Dutch version of the Geriatric Oral Health Assessment Index (GOHAI-NL) in care-dependent and care-independent older people
Source: BMC Geriatr. 2016 Feb 29;16:53. doi: 10.1186/s12877-016-0227-0 (PMC4772292; doi:10.1186/s12877-016-0227-0)
Supplement: Additional file 1: — GOHAI-NL. (DOCX 11 kb) [file 12877_2016_227_MOESM1_ESM.docx]

**Appendix 1. GOHAI-NL**

***1.***Hoe vaak hebt u de keuze van wat u eet, of de hoeveelheid die u eet, beperkt vanwege problemen met uw tanden of kunstgebit?

*□* nooit  ⁪ *□* zelden  ⁪ *□* af en toe   ⁪ *□* vaak   ⁪ *□* zeer vaak of altijd

***2.***Hoe vaak hebt u last gehad bij het bijten of kauwen van eten, zoals taai vlees of appels?

*□* nooit  ⁪ *□* zelden  ⁪ *□* af en toe   ⁪ *□* vaak   ⁪ *□* zeer vaak of altijd

***3.***Hoe vaak hebt u met gemak uw eten kunnen doorslikken?

*□* nooit  ⁪ *□* zelden  ⁪ *□* af en toe   ⁪ *□* vaak   ⁪ *□* zeer vaak of altijd

***4.***Hoe vaak hebt u uw tanden of kunstgebit als een probleem ervaren bij het spreken?

*□* nooit  ⁪ *□* zelden  ⁪ *□* af en toe   ⁪ *□* vaak   ⁪ *□* zeer vaak of altijd

***5.***Hoe vaak hebt u zonder ongemak kunnen eten wat u wilde?

*□* nooit  ⁪ *□* zelden  ⁪ *□* af en toe   ⁪ *□* vaak   ⁪ *□* zeer vaak of altijd

***6.***Hoe vaak hebt u uw contact met anderen beperkt door de conditie van uw tanden of kunstgebit?

*□* nooit  ⁪ *□* zelden  ⁪ *□* af en toe   ⁪ *□* vaak   ⁪ *□* zeer vaak of altijd

***7.*** Hoe vaak was u tevreden of blij met hoe uw tanden, tandvlees of kunstgebit eruit zien?

*□* nooit  ⁪ *□* zelden  ⁪ *□* af en toe   ⁪ *□* vaak   ⁪ *□* zeer vaak of altijd

***8.***Hoe vaak hebt u medicijnen gebruikt tegen pijn of ongemak in het gebied van uw mond?

*□* nooit  ⁪ *□* zelden  ⁪ *□* af en toe   ⁪ *□* vaak   ⁪ *□* zeer vaak of altijd

***9.*** Hoe vaak hebt u zich zorgen gemaakt om problemen met uw tanden, tandvlees of kunstgebit?

*□* nooit  ⁪ *□* zelden  ⁪ *□* af en toe   ⁪ *□* vaak   ⁪ *□* zeer vaak of altijd

***10.***Hoe vaak voelde u zich nerveus of in verlegenheid gebracht door problemen met uw tanden, tandvlees of kunstgebit?

*□* nooit  ⁪ *□* zelden  ⁪ *□* af en toe   ⁪ *□* vaak   ⁪ *□* zeer vaak of altijd

***11.***Hoe vaak hebt u zich ongemakkelijk gevoeld bij het eten in gezelschap van anderen door problemen met uw tanden of kunstgebit?

*□* nooit  ⁪ *□* zelden  ⁪ *□* af en toe   ⁪ *□* vaak   ⁪ *□* zeer vaak of altijd

***12.*** Hoe vaak waren uw tanden of uw tandvlees gevoelig voor hitte, kou of snoep?

*□* nooit  ⁪ *□* zelden  ⁪ *□* af en toe   ⁪ *□* vaak   ⁪ *□* zeer vaak of altijd
